# Distal humerus hemiarthroplasty for complex intra-articular fractures in elderly or nonfixable patients: a meta-analysis of clinical and functional outcomes

**DOI:** 10.1016/j.jseint.2026.101695

**Published:** 2026-03-11

**Authors:** Cerise Gosselin, Kévin A. Hao, Nicolas Bonnevialle, Pierre Mansat, Hugo Barret

**Affiliations:** a Centre Hospitalier Universitaire de Purpan, Toulouse, France; b Department of Orthopaedic Surgery & Sports Medicine, University of Florida, Gainesville, FL, USA

**Keywords:** Distal humerus fractures, Hemiarthroplasty, Elbow replacement, Meta-analysis, Complications, Elbow fracture, Elderly

## Abstract

**Background:**

Distal humerus fractures are difficult to treat when fixation is not possible, particularly in elderly patients. While total elbow arthroplasty is often used in elderly patients, it is generally not suitable for younger or more active individuals. Distal humerus hemiarthroplasty (DHH) may represent a viable alternative in selected cases. This systematic review and meta-analysis aimed to evaluate the clinical and functional outcomes of DHH in patients with complex distal humerus fractures.

**Methods:**

A systematic review was conducted in accordance with Preferred Reporting Items for Systematic Reviews and Meta-Analyses (PRISMA) guidelines. PubMed, Embase, and Cochrane Library were searched up to January 2024. Studies reporting outcomes of DHH for distal humerus fractures were included. Functional scores, range of motion, complications, and revision rates were pooled using a random-effects meta-analysis. Subgroup analyses were performed according to fracture type (Arbeitsgemeinschaft für Osteosynthesefragen classification) and surgical approach (triceps-on vs. triceps-off).

**Results:**

Nineteen studies including 593 patients (mean age 68 years; mean follow-up 53 months) were analyzed. The pooled Mayo Elbow Performance Score was 84.7 [95% confidence interval: 80.8-88.5], and the Disabilities of the Arm, Shoulder and Hand score was 20.8 [13.6-28.0]. The mean flexion–extension arc was 102° [97-106], and the pronation–supination arc was 155° [147-162]. The overall complication rate was 26.7% [18.3-37.1%], the reoperation rate 11.6% [5.3-23.4%], and the revision rate 3.4% [1.1%-10.1%]. Subgroup analyses showed comparable outcomes across fracture types, but a significantly higher instability rate in type B3 fractures (*P* = .017). No significant differences were found between triceps-on and triceps-off approaches.

**Conclusion:**

DHH yields satisfactory functional outcomes and range of motion in complex distal humerus fractures not amenable to fixation, particularly in elderly patients. However, a complication rate of 26.7% was observed. Careful patient selection remains essential, especially in fractures at high risk of post-operative instability.

Fractures of the distal humerus represent a complex therapeutic challenge due to the intricate anatomy of the elbow and the need for precise reconstruction to restore function. While open reduction and internal fixation (ORIF) remains the gold standard treatment,^3^^,^^14^^,^^39^^,^^48^ certain fracture patterns, particularly those with extensive comminution, osteoporotic bone, or loss of structural support, are not amenable to stable fixation. In such cases, fixation failure, hardware prominence, and residual stiffness are common.^12^^,^^28^^,^^37^^,^^46^ Total elbow arthroplasty (TEA) has emerged as an alternative in elderly patients, offering reliable outcomes when reconstruction is not feasible. However, TEA carries significant limitations, including lifelong activity restrictions, risk of polyethylene wear and loosening, and potential complications related to component fixation.^10^^,^^11^^,^^21^ These drawbacks make TEA unsuitable for younger, more active individuals.^4^^,^^16^^,^^19^^,^^23^^,^^25^^,^^33^

Distal humerus hemiarthroplasty (DHH) has gained attention as a middle ground, aiming to preserve functional activity while avoiding the constraints of a total implant.^1^^,^^8^ DHH replaces only the distal humeral articular surface, leaving the ulnar cartilage and soft tissues intact. While promising,^2^^,^^30^ this technique presents specific challenges such as joint instability and ulnar cartilage wear.^43^^,^^44^ Appropriate patient selection and surgical technique are therefore crucial to optimize outcomes.

Several case series and cohort studies have reported satisfactory functional results with DHH in both primary and salvage indications. However, outcomes vary widely across the literature, and the true complication and revision rates remain poorly defined. A previous systematic review by Kwak et al^24^ reported encouraging short-term outcomes, but subgroup analyses by fracture type or surgical approach were not performed, and several important studies have since been published.

Despite growing literature, no previous meta-analysis has pooled functional and complication outcomes while comparing subgroups by fracture pattern or surgical approach. Moreover, the heterogeneity of reported results highlights the need for a comprehensive synthesis of current evidence.

The objective of this meta-analysis is to evaluate the clinical and functional outcomes of distal humerus hemiarthroplasty for unreconstructable fractures, with specific focus on complication and revision rates, and subgroup comparisons based on fracture classification and surgical approach.

## Methods

### Study design

This systematic review and meta-analysis was conducted according to the Preferred Reporting Items for Systematic Reviews and Meta-Analyses (PRISMA) guidelines.^32^

### Data sources and search strategy

A comprehensive literature search was performed using PubMed, Embase, and the Cochrane Library from inception to January 2024. Search terms included “hemiarthroplasty,” “distal humerus fractures,” “elbow,” “outcomes,” and “complications.” The detailed search strategy for PubMed used the following Boolean combination of keywords and Medical Subject Heading terms: ("hemiarthroplasty" OR "partial arthroplasty") AND ("distal humerus" OR "elbow") AND ("fracture" OR "trauma") AND ("outcome" OR "result" OR "complication").

### Inclusion and exclusion criteria

Studies were eligible for inclusion if they were randomized controlled trials (RCTs), cohort studies, or case series involving patients with distal humerus fractures treated with hemiarthroplasty, and if they reported at least one clinical outcome such as range of motion, functional score, or complication/revision data. Studies were excluded if they were systematic reviews, meta-analyses, case reports, in vitro or biomechanical studies, or if they provided insufficient data on outcomes or complications.

### Data extraction and quality assessment

Two reviewers (CG, HB) independently screened titles and abstracts for eligibility. Full texts were reviewed for final inclusion. Disagreements were resolved by consensus or adjudication by a third reviewer (PM). Reviewers were not blinded to study authors or institutions.

Extracted data included study design, sample size, patient demographics, fracture classification (according to Arbeitsgemeinschaft für Osteosynthesefragen [AO]/OTA),^29^ implant type, surgical approach, range of motion, functional scores (Disabilities of the Arm, Shoulder and Hand [DASH],^18^ Mayo Elbow Performance Score [MEPS]^5^), pain (visual analog scale^36^), complications (eg, infection, loosening, instability, neuropathy, stiffness), and reoperation/revision rates.

Surgical approaches were grouped as described by Luciani et al^26^ into triceps-sparing approaches and triceps-off approaches. Triceps-sparing approaches (eg, paratricipital, triceps split, triceps reflecting, triceps-reflecting anconeus pedicle, lateral paraolecranon) aim to preserve the extensor mechanism and may facilitate early rehabilitation and reduce post-operative weakness. In contrast, triceps-off approaches (eg, V-Y lengthening, triceps tongue, olecranon osteotomy) provide greater exposure but may be associated with longer recovery, higher morbidity, and hardware-related complications.

### Risk of bias and study quality assessment

The quality of included studies was assessed using the Newcastle–Ottawa Scale (NOS) for nonrandomized studies and the Cochrane Risk of Bias Tool for RCTs. Most cohort studies demonstrated moderate to high quality, with a mean NOS score of 6.5/9. No funnel plot or Egger test was performed due to the substantial heterogeneity across outcomes and the limited number of studies per comparison.

### Statistical analysis

Data were synthesized descriptively and pooled using meta-analysis when at least 3 studies reported compatible outcomes (with sample size, mean, and standard deviation). A random-effects model was used a priori due to anticipated clinical and methodological heterogeneity.^6^ Forest plots were generated for each outcome.

Heterogeneity was quantified using the I^2^ statistic and between-study variance (τ^2^). The 95% prediction interval was calculated to estimate the range of true effects across comparable settings. All analyses were conducted using the metafor package in R software (version 4.2.0; R Core Team).^47^ Statistical significance was set at *P* < .05.

No formal correction for multiple comparisons (such as Bonferroni or Benjamini–Hochberg adjustment) was applied, as the subgroup analyses were exploratory and not intended for confirmatory statistical inference.

## Results

### Final article selection

**Figure 1:**
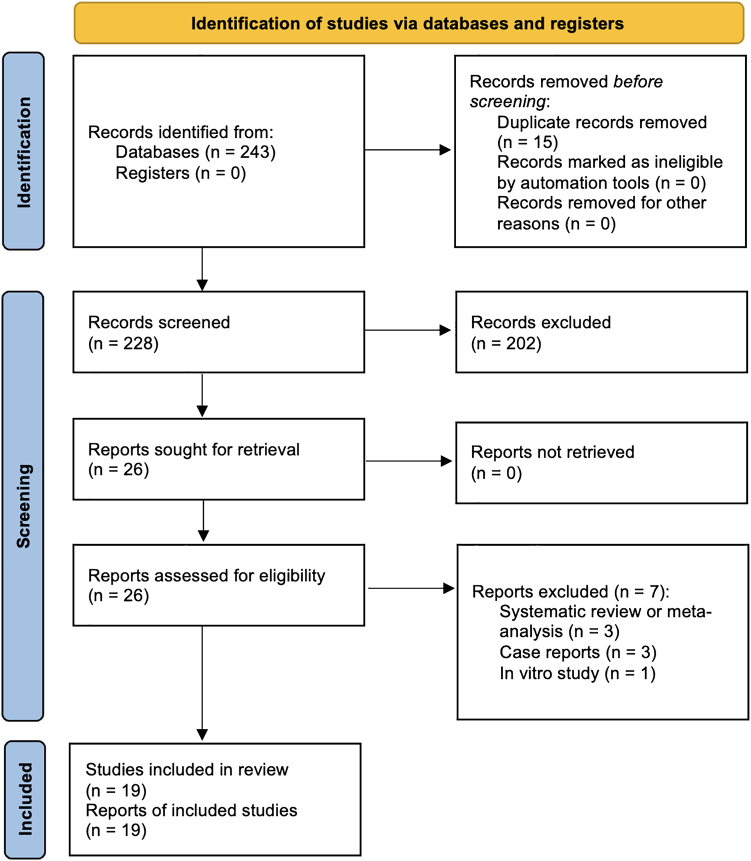
PRISMA flow chart. *PRISMA*, Preferred Reporting Items for Systematic Reviews and Meta-Analyses.

The initial search yielded 243 articles. After removal of duplicates and screening of titles and abstracts, 26 studies underwent full-text review. Of these, 7 were excluded (3 systematic reviews, 3 case reports, and 1 in vitro study), leaving 19 studies for inclusion in the meta-analysis. The PRISMA flowchart is shown in Fig. 1.

### Studies included

The 19 included studies represented a total of 593 patients (Table I), with a mean follow-up of 53 ± 28 months (range, 12-115 months). The average patient age was 68 ± 8 years (range, 44-79), and most studies involved elderly populations. All included studies were retrospective. Most implants used were the Tornier Latitude and Kudo Biomet prostheses. The methodological quality was moderate to high, with a mean NOS score of 6.5 out of 9.

**Table I:** Studies included in the meta-analysis.

Article	Number of patients (n)	Male (%)	Female (%)	Age mean (minimum–maximum)	FU mo (minimum–maximum)	Implant
Rotini et al, 2023	27	22%	78%	64 (45-78)	82 (12-151)	Tornier Latittude
Taylor et al, 2023	293	14%	86%	68 (NC-NC)	56 (NC-NC)	NC
Jenkins et al, 2022	37	30%	70%	75 (29-93)	61 (24-105)	Tornier Latittude
Schultzel et al, 2022	10	0%	0%	72 (56-81)	115 (96-144)	Tornier Latittude
Taylor et al, 2021	8	0%	100%	72 (63-83)	30 (11-59)	Tornier Latittude
Nestorson et al, 2015	42	7%	93%	72 (56-84)	34 (24-61)	Tornier Latittude
Adolfsson et al, 2012	8	0%	100%	79 (71-89)	54 (30-72)	Kudo Biomet
Heikink et al, 2015	6	0%	100%	69 (55-77)	54 (21-76)	Tornier Latittude
Phadnis et al, 2015	16	19%	81%	79 (60-90)	35 (24-79)	Tornier Latittude
Hohman et al, 2014	7	29%	71%	64 (33-75)	36 (26-60)	Tornier Latittude
Stephens et al, 2020	9	0%	0%	71 (55-92)	44 (23-96)	Tornier Latittude
Celli et al, 2022	41	20%	80%	63 (45-81)	92 (24-151)	Tornier Latittude
Al-Hamdani et al, 2019	24	13%	88%	65 (47-80)	20 (12-70)	Tornier Latittude
Persons et al, 2005	8	0%	0%	61 (46-83)	NC	Sorbie Wright
Burkhat et al, 2011	10	0%	100%	75 (62-88)	12 (6-23)	Tornier Latittude
Argintar et al, 2012	10	10%	90%	73 (56-77)	12 (NC-NC)	Tornier Latittude
Smith et al, 2013	26	12%	88%	60 (29-85)	80 (25-133)	Tornier + Sorbie
Smith et al, 2016	6	0%	100%	44 (29-52)	81 (24-133)	Tornier + Sorbie
Ricon et al, 2021	5	0%	100%	74 (66-83)	60 (24-96)	Kudo Biomet

*FU*, follow-up; NC, not reported.

### Clinical outcomes

**Figure 2:**
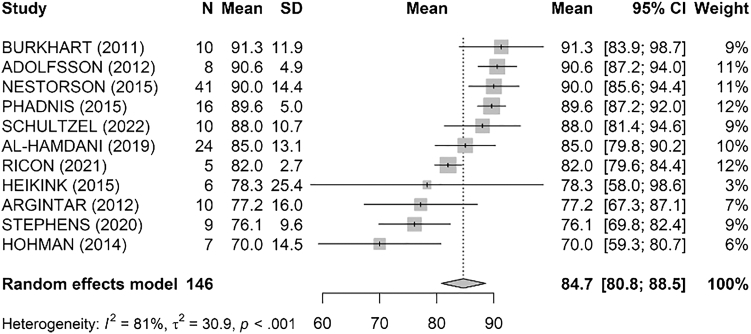
Pooled analysis of MEPS. *MEPS*, Mayo Elbow Performance Score; *SD*, standard deviation; *CI*, confidence interval.

Table II reports the main functional outcomes of the analyzed studies. The pooled analysis of MEPS across studies (Fig. 2) indicated a mean score of 84.7 [80.8; 88.5] with significant heterogeneity (I^2^ = 81%, τ^2^ = 30.9, *P* 0 < .001). The highest mean MEPS was reported by Burkhart et al^8^ with a score of 91.3, while the lowest was observed in the study by Hohman et al^17^ with a mean score of 70.

**Table II:** Functional outcomes in the studies included.

Article	Patients (n)	DASH (mean ± SD)	MEPS (mean ± SD)	Flexion (mean ± SD)	Extension loss (mean ± SD)	Arc FE (mean ± SD)	Supination (mean ± SD)	Pronation (mean ± SD)	Arc PS (mean)
Rotini et al, 2023	27	12.6 ± 8.9	89.3 ± NC	121 ± 15	25 ± 15	95 ± 24	76 ± 9	77 ± 9	153 ± 12
Taylor et al, 2023	293	NC	NC	NC	NC	NC	NC	NC	NC
Jenkins et al, 2022	37	14.9 ± NC	NC	132 + NC	19 ± NC	108 ± NC	81 ± NC	74 ± NC	155 ± NC
Schultzel et al, 2022	10	37.1 ± 18.4	88 ± 10.7	126 ± 33	36 ± 17	84 ± 34	66 ± 8	64 ± 15	130 ± 19
Taylor et al, 2021	8	NC	88.3 ± 5.8	135 ± 9	21 ± 15	114 ± NC	84 ± 8	87 ± 5	171 ± NC
Nestorson et al, 2015	42	20 ± 18.1	90 ± 14.4	127 ± 13	23 ± 17	103 ± 27	77 ± 13	77 ± 12	155 ± 25
Adolfsson et al, 2012	8	NC	90.6 ± 4.9	126 ± 7	31 ± 12	96 ± 17	80 ± NC	80 ± NC	NC
Heikink et al, 2015	6	18.48 ± 22.83	78.3 ± 25.43	122 ± 10	27 ± 8	96 ± 17	80 ± 8	84 ± 7	165 ± 14
Phadnis et al, 2015	16	11.2 ± 5.8	89.6 ± 5	131 ± 12	15 ± 9	116 ± 14	85 ± 4	85 ± 5	172 ± 9
Hohman et al, 2014	7	43 ± 25.1	70 ± 14.5	120 ± 14	19 ± 10	96 ± 22	73 ± 10	87 ± 8	160 ± 14
Stephens et al, 2020	9	NC	76.11 ± 9.6	NC	NC	NC	NC	NC	NC
Celli et al, 2022	41	15.9 ± 12.5	78.1 ± NC	119 ± 17	22 ± 15	97 ± 17	77 ± 10	77 ± 8	154 ± 16
Al-Hamdani et al, 2019	24	NC	85 ± 13.1	NC	NC	110 ± 22	NC	NC	160 ± 14
Persons et al, 2005	8	NC	NC	126 ± NC	22 ± NC	NC	NC	NC	NC
Burkhart et al, 2011	10	11.5 ± 10.6	91.3 ± 11.9	124 ± 14	17 ± 9	107 ± 15	79 ± 12	80 ± 10	160 ± 18
Argintar et al, 2012	10	35.1 ± 24	77.2 ± 16	121 ± 32	22 ± 16	102 ± 25	69 ± 6	64 ± 11	133 ± 15
Smith et al, 2013	26	19.0 ± NC	90.4 ± NC	133 ± NC	17 ± NC	116 ± NC	83 ± NC	83 ± NC	165 ± NC
Smith et al, 2016	6	12 ± 11	88 ± 17.5	126 ± 9	23 ± 15	103 ± 19	71 ± 40	78 ± 28	149 ± 69
Ricon et al, 2021	5	NC	82 ± 2.7	109 ± 9	8 ± 4	100 ± 7	NC	NC	NC

*DASH*, Disabilities of the Arm, Shoulder and Hand; *FE,* flexion–extension; *MEPS*, Mayo Elbow Performance Score; *NC,* not reported; *PS*, pronation–supination; *SD*, standard deviation.

**Figure 3:**
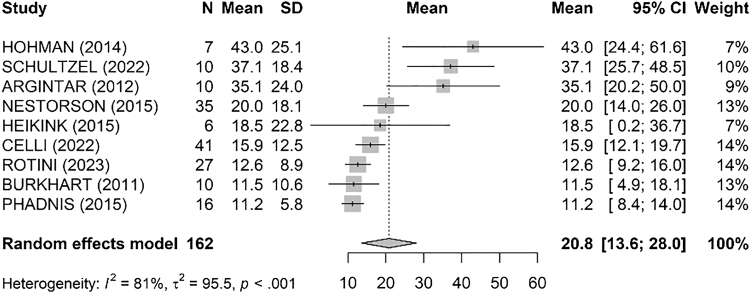
Pooled analysis of DASH scores. *DASH*, Disabilities of the Arm, Shoulder and Hand; *SD*, standard deviation; *CI*, confidence interval.

The pooled analysis of DASH scores (Fig. 3) resulted in a mean of 20.8 [13.6; 28.0] with significant heterogeneity (I^2^ = 81%, τ^2^ = 95.5, *P* 0 < .001). The highest mean DASH score was reported by Hohman et al^17^ at 43.0, indicating greater disability, while the lowest was by Phadnis et al^34^ at 11.2, indicating less disability.

**Figure 4:**
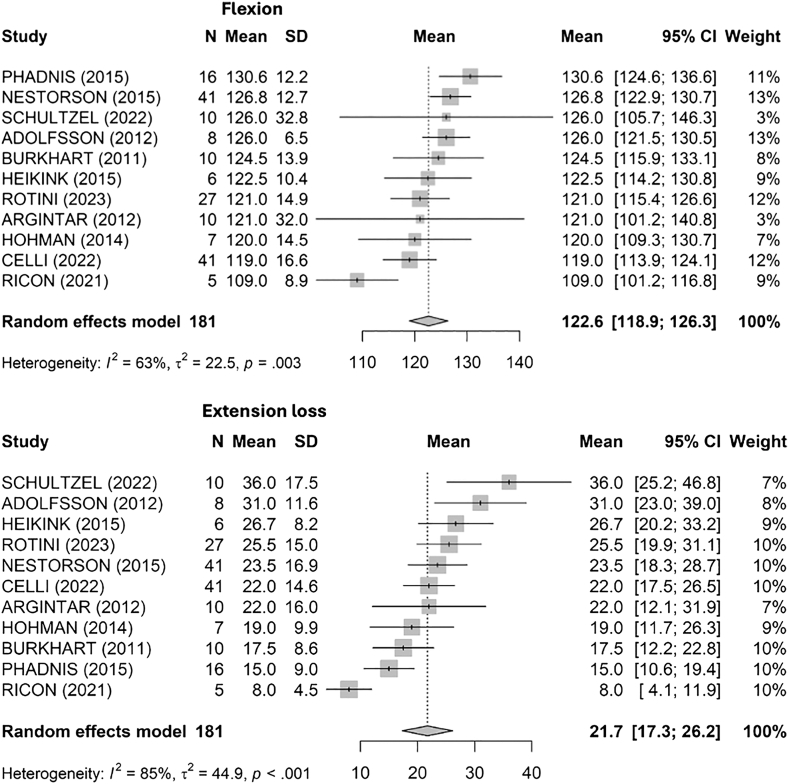
Flexion degrees and extension loss at final follow up across studies. *SD*, standard deviation; *CI*, confidence interval.

Flexion degrees were assessed across multiple studies (Fig. 4), yielding a pooled mean flexion of 123° [119; 126] (I^2^ = 63%, τ^2^ = 22.5, *P* = .003). The highest mean flexion was recorded by Phadnis et al^34^ at 131°, whereas the lowest was noted by Ricon et al^38^ with a mean of 109°.

Extension loss was evaluated (Fig. 4), showing a pooled mean of 22° [17; 26] with substantial heterogeneity (I^2^ = 85%, τ^2^ = 44.9, *P* 0 < .001). The greatest mean extension loss was reported by Schultzel et al^41^ at 36°, and the lowest by Ricon et al^38^ at 8°.

**Figure 5:**
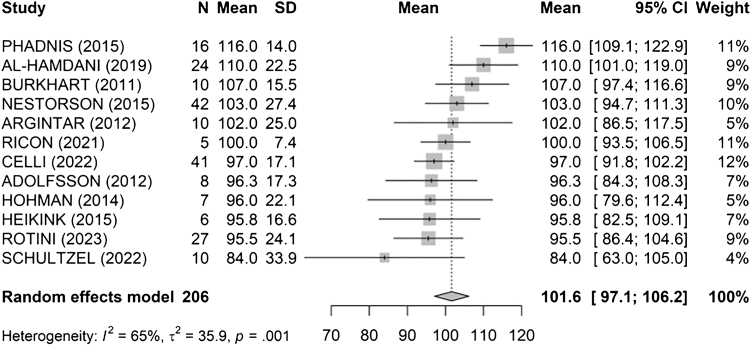
Overall mean arc of flexion and extension. *SD*, standard deviation; *CI*, confidence interval.

The overall mean arc of flexion and extension (Fig. 5) was calculated to be 102° [97; 106] (I^2^ = 65%, τ^2^ = 35.9, *P* = .001). The study by Phadnis et al^34^ recorded the highest mean arc at 116°, while the lowest was by Schultzel et al^41^ at 84°.

**Figure 6:**
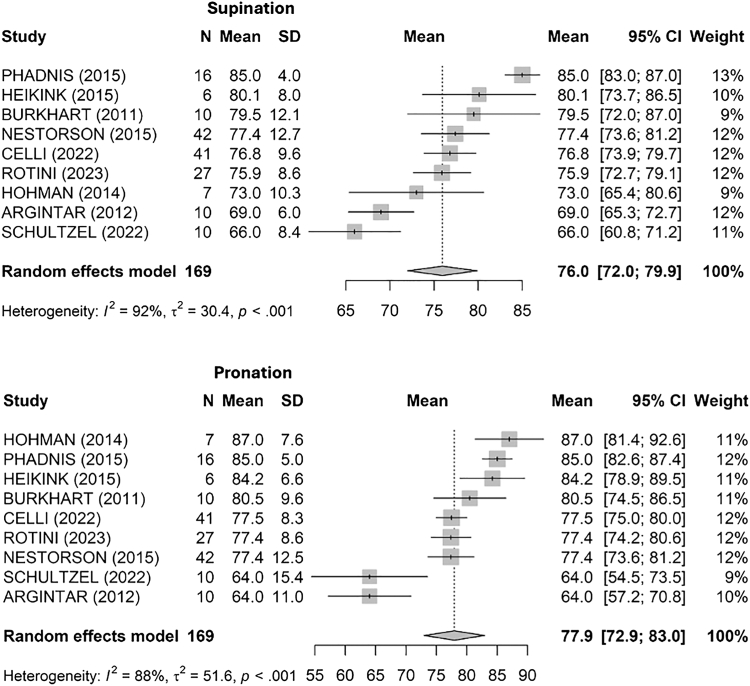
Pooled supination and pronation at final follow-up. *SD*, standard deviation; *CI*, confidence interval.

Supination ability (Fig. 6), measured in degrees, resulted in a pooled mean of 76° [72; 80] (I^2^ = 92%, τ^2^ = 30.4, *P* < .001). The highest mean supination was reported by Phadnis et al^34^ at 85°, and the lowest by Schultzel et al^41^ at 66°.

Pronation degrees (Fig. 6) showed a pooled mean of 78° [73; 83] with significant heterogeneity (I^2^ = 88%, τ^2^ = 51.6, *P* < .001). Hohman et al^17^ reported the highest mean pronation at 87°, while the lowest was seen in Argintar et al^4^ and Schultzel et al,^41^ both at 64°.

**Figure 7:**
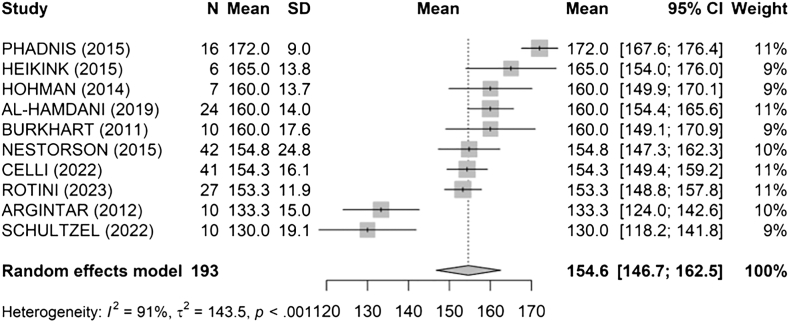
Pooled arc of pronation and supination at final follow-up. *SD*, standard deviation; *CI*, confidence interval; *AO,* Arbeitsgemeinschaft für Osteosynthesefragen.

The overall mean arc of pronation and supination (Fig. 7) was 155° [147; 162] (I^2^ = 91%, τ^2^ = 143.5, *P* < .001). Phadnis et al^34^ had the highest mean arc at 172°, whereas Schultzel et al^41^ recorded the lowest at 130°.

### Complications and reoperations

**Figure 8:**
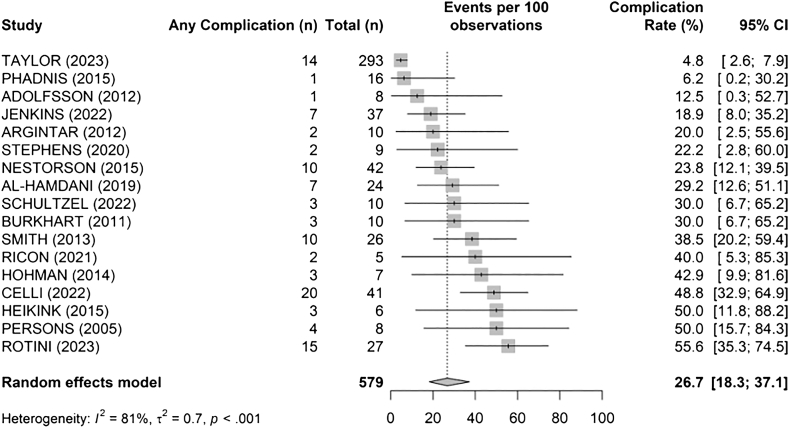
Overall complication rate across studies. *CI*, confidence interval.

The overall complication rate was 26.7% (95% confidence interval [CI]: 18.3%-37.1%), with high heterogeneity (I^2^ = 81%, τ^2^ = 0.7, *P* < .001) (Fig. 8, Table II). The most frequent complications were post-operative stiffness (11%), transient ulnar neuropathy (7%), and joint instability (5%). Rates varied across studies, ranging from 4.8% to 55.6%.

**Table III:** Complications rates and types of complications in the different studies.

Study	Number of patients (n)	Complication rate (%)	Types of complications
Rotini et al, 2023	27	55%	2 ulnar impingement, 7 stiffness, 5 ulnar nerve neuropathy, 1 instability
Taylor et al, 2023	293	18%	4 infections, 1 fracture, 4 loosening, 5 instability
Jenkins et al, 2022	37	19%	1 traumatic dislocation, 1 wound breakdown, 1 stiffness, 1 ulnar nerve neuropathy, 3 instability
Schultzel et al, 2022	10	30%	1 radial nerve palsy, 1 olecranon hardware discomfort, 1 fracture
Taylor et al, 2021	8	25%	1 traumatic dislocation, 1 ulnar nerve neuropathy
Nestorson et al, 2015	42	24%	1 wound breakdown, 4 stiffness, 3 ulnar nerve neuropathy, 1 loosening, 1 instability
Adolfsson et al, 2012	8	12%	1 fracture
Heikink et al, 2015	6	50%	2 ulnar nerve neuropathy, 3 instability
Phadnis et al, 2015	16	6%	1 ulnar nerve neuropathy
Hohman et al, 2014	7	43%	1 ulnar nerve neuropathy, 2 olecranon hardware discomfort
Stephens et al, 2020	9	22%	1 wound breakdown, 1 ulnar nerve neuropathy
Celli et al, 2022	41	45%	10 stiffness, 6 ulnar nerve neuropathy, 4 instability
Al-Hamdani et al, 2019	24	29%	3 stiffness, 3 ulnar nerve neuropathy
Persons et al, 2005	8	50%	1 ulnar impingement, 1 ulnar nerve neuropathy, 3 olecranon hardware discomfort
Burkhart et al, 2011	10	30%	1 triceps weakness, 1 wound breakdown, 1 ulnar nerve neuropathy
Argintar et al, 2012	10	20%	1 ulnar nerve neuropathy, 1 olecranon hardware discomfort
Smith et al, 2013	26	39%	1 wound breakdown, 1 stiffness, 4 ulnar nerve neuropathy, 2 fracture, 2 loosening
Smith et al, 2016	6	33%	1 stiffness, 2 loosening
Ricon et al, 2021	5	40%	1 wound breakdown, 1 ulnar nerve neuropathy

**Figure 9:**
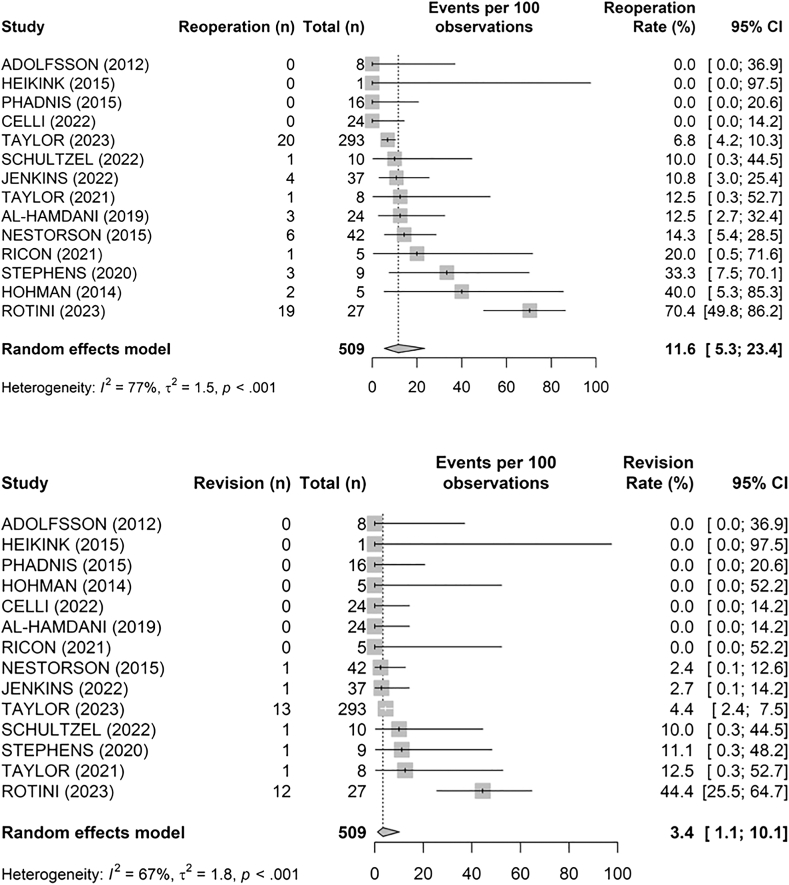
Pooled revision and reoperation rates at final follow-up. *CI*, confidence interval.

The pooled reoperation rate was 11.6% (95% CI: 5.3%-23.4%), while the revision rate was 3.4% (95% CI: 1.1%-10.1%) (Fig. 9, Table IV). Notably, some studies reported high rates of reoperation due to secondary arthrolysis or conversion to TEA (eg, Rotini et al^39^ reported a 70% reoperation rate).

**Table IV:** Reoperation rates and types of reoperations among studies.

Study	Revision rate (%)	Types of revisions
Rotini et al, 2023	70%	7 arthrolysis, 12 TEA conversion
Taylor et al, 2023	7%	13 TEA conversion
Jenkins et al, 2022	11%	1 ulnar nerve neurolysis, 1 wound débridement, 1 arthrolysis, 1 TEA conversion
Schultzel et al, 2022	10%	1 olecranon hardware removal
Taylor et al, 2021	12%	1 TEA conversion
Nestorson et al, 2015	14%	1 LCL reconstruction, 4 HO excision, 1 TEA conversion
Adolfsson et al, 2012	0%	
Heikink et al, 2015	0%	
Phadnis et al, 2015	0%	
Hohman et al, 2014	28%	2 olecranon hardware removal, 1 ulnar nerve transposition
Stephens et al, 2020	33%	1 ulnar nerve neurolysis, 1 wound débridement, 1 TEA conversion
Celli et al, 2022	2%	1 conversion TEA
Al-Hamdani et al, 2019	12%	3 arthrolysis
Persons et al, 2005	62%	4 olecranon hardware removal, 1 TEA conversion
Burkhart et al, 2011	20%	1 wound débridement, 1 TEA conversion
Argintar et al, 2012	10%	1 olecranon hardware removal
Smith et al, 2013	38%	4 ulnar nerve transposition, 1 wound débridement, 1 arthrolysis, 4 TEA conversion
Smith et al, 2016	66%	4 olecranon hardware removal, 1 arthrolysis, 2 TEA conversion
Ricon et al, 2021	20%	1 olecranon hardware removal

*FE, *flexion-extension*; HO, *heterotopic ossification*; LCL, *lateral collateral ligament*; TEA*, total elbow arthroplasty.

### Subgroup analyses

#### By fracture type (AO classification)

Subgroup analysis by fracture pattern (B1/B2, B3, C2/C3) is shown in Table V, including 16, 53, and 141 patients, respectively. No significant differences were observed in MEPS, DASH, or range of motion among the groups. However, the rate of post-operative instability was significantly higher in B3 fractures compared to C2/C3 (12% vs. 1%, *P* = .017). Given the small number of patients in some subgroups, these findings should be interpreted with caution.

**Table V:** Comparison of clinical outcomes based on fracture pattern.

Outcome measure	B1/B2		B3		C2/C3		*P* value
	N	Mean ± SD	N	Mean ± SD	N	Mean ± SD	
Age at surgery (yr)	16	70.9 ± 7.8	53	65.9 ± 9.0	141	68.8 ± 11.9	.077
Female sex	0	NA	44	82% (36)	76	91% (69)	.164
Outcome scores							
MEPS	16	92.2 ± 14.4	19	86.8 ± 16.5	119	86.0 ± 13.3	.294
DASH score	15	14.3 ± 17.4	49	15.0 ± 12.6	100	21.2 ± 18.9	.056
ROM							
Flexion (°)	16	130 ± 15	53	125 ± 15	115	124 ± 17	.401
Extension loss (°)	16	23 ± 12	53	21 ± 15	115	23 ± 15	.594
FE arc (°)	16	107 ± 21	53	104 ± 24	140	101 ± 24	.544
Pronation (°)	16	79 ± 3	50	78 ± 10	105	78 ± 15	.722
Supination (°)	16	79 ± 3	50	77 ± 11	105	77 ± 17	.169
PS arc (°)	16	158 ± 4	50	155 ± 17	129	154 ± 28	.206
Complications							
Overall	0	NA	42	48% (20)	82	32% (26)	.116
Triceps weakness	0	NA	42	0% (0)	82	1% (1)	1.000
Implant loosening	0	NA	42	0% (0)	83	1% (1)	1.000
Wound necrosis	0	NA	42	0% (0)	82	1% (1)	1.000
Infection	0	NA	42	0% (0)	82	2% (2)	.548
Post-operative stiffness	0	NA	42	21% (9)	82	12% (10)	.195
Ulnar neuropathy	0	NA	42	21% (9)	82	12% (10)	.195
Olecranon hardware discomfort	0	NA	42	0% (0)	82	4% (3)	.550
Instability	0	NA	42	12% (5)	82	1% (1)	**.017**

Bold values indicate statistically significant differences (*P* < .05).

*DASH*, Disabilities of the Arm, Shoulder and Hand; *MEPS*, Mayo Elbow Performance Score; *PS,* pronation–supination; *ROM*, range of motion; *SD*, standard deviation.

#### By surgical approach (triceps on vs. off)

Outcomes based on surgical approach are presented in Table VI, with 168 patients operated using a triceps-on and 78 using a triceps-off technique. No statistically significant differences were found in functional outcomes or range of motion between the 2 groups. The overall complication rate was higher in the triceps-on group (42% vs. 28%), but this difference did not reach statistical significance (*P* = .134). As the distribution of cases between approaches was uneven, subgroup comparisons should also be interpreted with caution.

**Table VI:** Comparison of clinical outcomes based on whether hemiarthroplasty was performed with the triceps left on or taken off.

Outcome measure	Triceps on		Triceps off		*P* value
	N	Mean ± SD	N	Mean ± SD	
Age at surgery (yr)	168	66.6 ± 11.1	78	70.5 ± 10.9	.009
Female sex	106	87% (92)	39	92% (36)	.561
Outcome scores					
MEPS	87	87.4 ± 15.2	77	84.8 ± 12.1	.241
DASH score	141	18.2 ± 16.3	42	21.4 ± 19.5	.334
ROM					
Flexion (°)	158	125 ± 16	45	125 ± 19	.999
Extension loss (°)	158	23 ± 15	45	20 ± 12	.161
Flexion/extension arc (°)	159	101 ± 25	69	105 ± 22	.213
Pronation (°)	146	78 ± 13	43	80 ± 12	.379
Supination (°)	146	77 ± 15	43	78 ± 11	.850
Pronation/supination src (°)	146	155 ± 26	67	156 ± 19	.774
Complications					
Overall	100	42% (42)	43	28% (12)	.134
Triceps weakness	100	0% (0)	43	2% (1)	.301
Implant loosening	101	1% (1)	43	0% (0)	1.000
Wound necrosis	100	1% (1)	43	0% (0)	1.000
Infection	100	1% (1)	43	2% (1)	.512
Postoperative stiffness	100	20% (20)	43	7% (3)	.080
Ulnar neuropathy	100	17% (17)	43	9% (4)	.307
Olecranon hardware discomfort	100	1% (1)	43	5% (2)	.215
Instability	100	7% (7)	43	2% (1)	.436

*DASH*, Disabilities of the Arm, Shoulder and Hand; *MEPS*, Mayo Elbow Performance Score; *ROM*, range of motion; *SD*, standard deviation.

## Discussion

This meta-analysis of 19 studies including 593 patients provides the most comprehensive synthesis to date of distal humerus hemiarthroplasty (DHH) outcomes. Functional results were satisfactory, with a mean MEPS of 84.7 and a mean DASH score of 20.8. Elbow flexion–extension and forearm rotation arcs exceeded functional thresholds, averaging 102° and 155°, respectively. The overall complication rate was 26.7%, with a revision rate of 3.4% and a reoperation rate of 11.6%. Subgroup analyses revealed higher instability rates in AO type B3 fractures and no significant differences in outcomes based on surgical approach (triceps sparing vs. triceps off).

Our findings align with earlier reports, while expanding upon them. Kwak et al^24^ analyzed 115 cases and found similar MEPS (85.8) and DASH (20) scores but did not stratify outcomes by fracture type or surgical technique. Dunn et al^13^ and Piggott et al^35^ also reported comparable motion arcs (93°-108° flexion–extension), but with less granularity. Piggott et al noted an overall reoperation rate of 17% and revision rate of 3%, both comparable to our pooled estimates. Recent data from Burden et al,^7^ comparing DHH and TEA in elderly patients, revealed similar MEPS scores (87 vs. 88) and DASH scores (20 vs. 38), suggesting functional equivalence but differing complication profiles.

DHH appears particularly useful in elderly or low-demand patients with unreconstructable distal humerus fractures, where internal fixation is unlikely to succeed.^2^^,^^9^^,^^24^^,^^31^^,^^39^ It offers faster post-operative mobilization and avoids polyethylene-related complications of TEA. In younger patients, DHH may preserve more bone stock and joint kinematics, although long-term durability remains unclear. Compared to TEA, it avoids lifetime activity restrictions, especially relevant for active or semiactive individuals in their 60s or younger. Proper indication remains critical: DHH is most appropriate when the humeral columns can be stabilized, cartilage wear risk is acceptable, and soft tissues are intact or reparable. Indeed, the repair of soft tissues, particularly the collateral ligaments, is critical to ensure elbow stability after DHH. Preservation or anatomical reconstruction of these ligaments should be considered mandatory whenever feasible.

Subgroup analysis based on AO fracture classification revealed that instability was significantly more frequent in type B3 fractures (12%) than in C2/C3 fractures (1%, *P* = .017). B3 fractures, involving isolated trochlear or capitellar fragments, lack columnar support and ligament insertions, which may explain the higher risk of instability. In contrast, C-type fractures often allow for reconstruction of both columns and ligamentous repair, providing greater prosthetic stability. Ensuring proper ligament reconstruction is critical.^22^^,^^27^^,^^45^ Techniques described by Phadnis et al^34^ involve anchoring the medial and lateral ligaments to the humeral shaft and securing the epicondyles around the implant with transosseous sutures. Accurate positioning of the prosthetic axis of rotation is also key to avoiding instability and wear.^40^ Intraoperative assessment of stability, preservation of the triceps (when possible), and appropriate implant sizing all contribute to outcome success. The comparison between triceps-sparing and triceps-off approaches in our study revealed no significant difference in functional outcomes or complication rates. While triceps-sparing techniques may facilitate earlier rehabilitation, the choice of approach should be tailored to fracture exposure requirements and surgeon experience.

Compared to ORIF, DHH avoids risks of nonunion, hardware failure, and stiffness, especially in elderly patients with poor bone quality. Yetter et al^49^ reported an ORIF complication rate of 53% and a reoperation rate of 21% across 2,362 elbows, substantially higher than with DHH in our review. TEA, although reliable in elderly patients, carries its own risks. Githens et al^15^ and Seok et al^42^ showed that TEA has a higher rate of minor complications and periprosthetic fractures but slightly fewer major complications than ORIF.

A recent multicenter RCT by Jonsson et al^20^ found no significant differences in functional scores between TEA and DHH at short-term follow-up. However, the TEA group had slightly better flexion–extension arcs (107° vs. 97°), while grip strength and DASH scores were similar. This trial supports the use of DHH as a valid alternative to TEA in carefully selected patients. Based on current evidence and clinical practice considerations, a simplified decision-making algorithm can be proposed (Table VII).

**Table VII:** Suggested treatment strategy for distal humerus fractures based on patient profile.

Patient profile	Fracture type	Bone quality	Functional demand	Preferred treatment	Comments
Young (<60 yr)	C2/C3 complex articular	Good	High	ORIF	Gold standard when fixation is possible
Young (<60 yr)	B3 or severe comminution	Moderate	High	DHH	Consider only if ORIF not feasible; repair ligaments carefully
Elderly (≥65 yr)	C2/C3	Poor	Low to moderate	TEA	Limited lifting post-operative; long-term concerns with implant survival
Elderly (≥65 y)	B3/column intact	Moderate	Moderate to high	DHH	Avoid TEA in active patients; assess risk of instability
Frail patient (>75 yr)	Any unreconstructable	Poor	Low	TEA	Preferred for rapid recovery and pain control
Highly active patient, any age	Coronal shear, simple B1	Good	High	ORIF	Consider arthroplasty only as salvage

*ORIF*, open reduction internal fixation; *TEA*, total elbow arthroplasty.

This table summarizes a proposed treatment algorithm for distal humerus fractures based on patient-specific factors including age, bone quality, fracture pattern, and functional demand. ORIF is favored in younger patients with good bone stock and reconstructable fractures. TEA is generally reserved for low-demand elderly patients with poor bone quality and nonreconstructable fractures. Distal humerus hemiarthroplasty (DHH) may be considered in active patients, especially when internal fixation is not feasible and TEA is contraindicated. Fracture patterns involving the capitellum or trochlea without column involvement (eg, AO type B3) may carry a higher risk of post-operative instability with DHH.

This study has several limitations. First, many included studies were retrospective, with variable quality and potential selection bias. The lack of uniform outcome reporting, heterogeneity in implant type and technique, and limited long-term follow-up reduce the generalizability of findings. In addition, the small number of RCTs and limited availability of comparative data restrict the strength of evidence in subgroup analyses. Lastly, no assessment of publication bias (eg, funnel plot) was performed due to the small number of studies per outcome. Moreover, most included studies had relatively short follow-up periods, often limited to 1 or two years. As progressive cartilage wear of the ulnohumeral joint may develop several years after implantation, long-term results could potentially reveal higher rates of degenerative changes or secondary conversions to TEA. Future studies with extended follow-up are therefore essential to assess the true durability of DHH.

Despite these limitations, this study presents the most extensive pooled analysis to date of DHH outcomes, with a rigorous methodology, subgroup analyses by fracture type and surgical approach, and comprehensive reporting of complications and revisions. The adherence to PRISMA standards enhances transparency and reproducibility.

Future research should focus on prospective cohort studies and RCTs comparing DHH to ORIF and TEA in specific populations. Standardized reporting of complications, functional scores, and long-term implant survival is essential. In addition, the role of ligament reconstruction techniques and implant design improvements (eg, anatomical offset, constraint options) should be evaluated to reduce instability and wear.

## Conclusion

While DHH provides satisfactory functional outcomes for unreconstructable distal humerus fractures, particularly in elderly patients, it remains associated with substantial complication and reoperation rates. Its indications should be carefully weighed, and future prospective, comparative studies are needed to clarify its place among available surgical options.

## Disclaimers:

Funding: No funding was disclosed by the authors.

Conflicts of interest: The authors, their immediate families, and any research foundations with which they are affiliated have not received any financial payments or other benefits from any commercial entity related to the subject of this article.

Given his role as Editor in Chief, Dr. Pierre Mansat had no involvement in the peer-review of this article and has no access to information regarding its peer-review. Full responsibility for the editorial process for this article was delegated to Dr. William J. Mallon.

## Declaration of generative AI and AI-assisted technologies

No generative AI and AI-assisted technologies were used in the writing process of this manuscript.
